# Salt intake and mental distress among rural community-dwelling Japanese men

**DOI:** 10.1186/s40101-015-0064-4

**Published:** 2015-06-25

**Authors:** Yuji Shimizu, Koichiro Kadota, Jun Koyamatsu, Hirotomo Yamanashi, Mako Nagayoshi, Miki Noda, Takayuki Nishimura, Jun Tayama, Yasuhiro Nagata, Takahiro Maeda

**Affiliations:** Department of Community Medicine, Nagasaki University Graduate School of Biomedical Science, Nagasaki, Japan; Department of Island and Community Medicine, Nagasaki University Graduate School of Biomedical Science, Nagasaki, Japan; Department of Public Health, Nagasaki University Graduate School of Biomedical Science, Nagasaki, Japan; Graduate School of Education, Nagasaki University, Nagasaki, Japan; Center for Health and Community Medicine, Nagasaki University, Nagasaki, Japan; Center for Comprehensive Community Care Education, Nagasaki University Graduate School of Biomedical Science, Nagasaki, Japan

## Abstract

**Background:**

Activated mineralocorticoid receptors influence the association between daily salt intake and blood pressure. A relatively low mineralocorticoid receptor function is reported to be a risk for mental distress such as depression. Since mental distress is also a known risk for hypertension and cardiovascular disease, understanding of the association between estimated daily salt intake and mental distress contributing to hypertension is important for risk estimation for cardiovascular disease. However, no single study has reported this association.

**Methods:**

We conducted a cross-sectional study of 1014 Japanese men undergoing general health check-ups. Mental distress was diagnosed as a Kessler 6 scale score ≥5. We also classified mental distress by levels of hypertension. Estimated daily salt intake was calculated from a causal urine specimen.

**Results:**

Independent from classical cardiovascular risk factors and thyroid disease, we found a significant inverse association between estimated daily salt intake and mental distress. When we analyzed for mental distress and hypertension, we also found a significant association. With the reference group being the lowest tertiles of estimated daily salt intake, the multivariable odds ratios (ORs) of mental distress and mental distress with hypertension for the highest tertiles were 0.50 (0.29–0.88) and 0.46 (0.22–0.96).

**Conclusions:**

Lower estimated daily salt intake is a significant risk of mental distress for rural community-dwelling Japanese men. Since depression is reported to be associated with cardiovascular disease, risk estimation for the lower intake of salt on mental distress, especially for mental distress with hypertension, may become an important tool to prevent cardiovascular disease.

## Introduction

Previous studies reported that increased salt sensitivity of blood pressure is a major contributing factor in a group of hypertensive subjects [1, 2]. However, there are individual differences in the salt sensitivity of blood pressure and kidney injury [3]. Recent studies also revealed the activation of two pathways (aldosterone/mineralocorticoid receptor pathway and renal sympathetic nervous system) in salt-sensitive hypertension. The mineralocorticoid and glucocorticoid receptors may contribute to impaired renal excretory function, resulting in salt-sensitive hypertension by increasing sodium reabsorption at different tubular segments [4].

Sodium reabsorption might also be correlated with psychological activity; inhibition of renal salt reabsorption is compensated for by stimulation of the salt appetite and vice versa [5]. Stress exposure activates the hypothalamic-pituitary-adrenal axis and results in the release of corticosteroids which bind to two types of receptors in the brain: the mineralocorticoid receptor and the glucocorticoid receptor. A previous study has reported that individuals with relatively low mineralocorticoid receptor function might possess increased susceptibility for depression from stress [6]. Thus, increasing the activity or expression of brain mineralocorticoid receptors may prevent or reverse the symptoms of stress-related depression.

In the studies that indicate that reducing salt intake is an efficient way of preventing the development of hypertension, there might be an underlying risk of mental distress. Since persons with depression are reported to be more likely to eventually develop cardiovascular disease and also have a higher mortality rate than the general population [7], risk estimation for mental distress by measuring salt intake may become an important tool to prevent cardiovascular disease.

Therefore, we hypothesized that independent from classical cardiovascular risk factors, lower salt intake might be associated with mental distress. To investigate those associations, we conducted a cross-sectional study of 1014 Japanese men who participated in general health check-up in 2014.

## Methods

### Study population

The original population included 1223 men 40 to 94 years old residing in rural communities in Nagasaki Prefecture in western Japan. Participants were recruited in 2014. A total of 209 individuals with missing data (including 1 individual without blood pressure data, 4 individuals without smoking status data, 14 individuals without Kessler 6 (K6) scale data, and 190 individuals without blood and urinary test data) were excluded, leaving 1014 men enrolled in this study. The mean age of the study sample was 65.1 ± 10.1 years old (range 40–94 years old). Written consent forms were available in Japanese to ensure comprehensive understanding of the study objectives, and informed consent was signed by the participants. This study was approved by the Ethics Committee for Human Use of Nagasaki University (project registration number 0501120073).

### Data collection and laboratory measurements

Systolic and diastolic blood pressures at rest in a sitting position were recorded using a blood pressure measuring device (HEM-907; Omron, Kyoto, Japan) by trained technicians. Hypertension was defined as systolic blood pressure ≥140 mmHg and/or diastolic blood pressure ≥90 mmHg and/or taking antihypertensive medication. Height in stocking feet and weight in light clothing were measured with an automatic body composition analyzer (BF-220; Tanita, Tokyo, Japan) before blood was drawn. Trained interviewers obtained information on smoking status, drinking status, medical history, and a questionnaire of mental stress named Kessler 6 was administered. The K6 was developed with the goal of being sensitive to the upper 90th to 99th percentile range of the population distribution of mental distress [8]. The current study examines the utility of the K6 scale for identifying mental distress at moderate levels. A study of 50,880 adult participants in the 2007 California Health Interview Survey reported that the receiver operating characteristic (ROC) curve analysis identified a K6 scale score ≥5 as optimal for identifying respondents with mental health treatment needs [9]. Therefore, we defined mental distress as a K6 scale score ≥5 in the present study.

Fasting blood samples were obtained with a sodium fluoride tube and a siliconized tube. Samples from the sodium fluoride tube were used to measure hemoglobin A1c (HbA1c), and samples from the siliconized tube were used for serum separation and centrifugation after blood coagulation to measure serum triglycerides (TG), HDL-cholesterol, aspartate transaminase (AST), and creatinine. Spot voiding urinary samples were also taken to measure the urinary concentration of sodium and creatinine. All measurements were determined by standard laboratory procedures at SRL, Inc. (Tokyo, Japan). We calculated estimated daily salt intake by following the published calculation formula [10]. The calculation of predicted values of 24-h urinary creatinine excretion (PRCr) used the formula:

$$ \mathrm{PRCr}\left(\mathrm{mg}/\mathrm{day}\right)=\hbox{--} 2.04\times \mathrm{age}+14.89 \times \mathrm{weight}\left(\mathrm{kg}\right)+16.14\times \mathrm{height}\left(\mathrm{cm}\right)\hbox{--} 2244.45 $$

$$ \mathrm{SUNa}=\mathrm{N}\mathrm{a}\ \mathrm{concentration}\ \mathrm{in}\ \mathrm{the}\ \mathrm{spot}\ \mathrm{voiding}\ \mathrm{urine}\ \left(\mathrm{mEq}/\mathrm{L}\right) $$

$$ \mathrm{SUCr}=\mathrm{Creatinine}\ \mathrm{concentration}\ \mathrm{in}\ \mathrm{the}\ \mathrm{spot}\ \mathrm{voiding}\ \mathrm{urine}\ \left(\mathrm{mg}/\mathrm{dL}\right) $$

XNa term is calculated from the following formula.

$$ \mathrm{X}\mathrm{N}\mathrm{a}=\mathrm{SUNa}/\mathrm{SUCr}\times \mathrm{PRCr} $$

$$ 24\mathrm{HuNaV}=\mathrm{Estimated}\ \mathrm{population}\ \mathrm{mean}\ \mathrm{levels}\ \mathrm{of}\ 24\hbox{-} \mathrm{h}\ \mathrm{urinary}\ \mathrm{sodium}\ \mathrm{excretion}\ \left(\mathrm{mEq}/\mathrm{day}\right) $$

$$ 24\mathrm{HuNaV}\ \left(\mathrm{mEq}/\mathrm{day}\right)=21.98\times \mathrm{X}\mathrm{N}{\mathrm{a}}^{0.392} $$

$$ \mathrm{Estimated}\ \mathrm{daily}\ \mathrm{salt}\ \mathrm{intake}\ \left(\mathrm{g}/\mathrm{day}\right) = 24\mathrm{HuNaV}\ \left(\mathrm{mEq}/\mathrm{day}\right) \times 0.0585 $$

The glomerular filtration rate (GFR) was estimated by using an established method with three variations recently proposed by a working group of the Japanese Chronic Kidney Disease Initiative [11], according to which:

$$ \mathrm{G}\mathrm{F}\mathrm{R}\ \left(\mathrm{ml}/ \min /1.73\ {\mathrm{m}}^2\right)=194 \times {\left(\mathrm{serum}\ \mathrm{creatinine}\ \left(\mathrm{enzyme}\ \mathrm{method}\right)\right)}^{\hbox{--} 1.094} \times {\left(\mathrm{age}\right)}^{\hbox{--} 0.287} $$

### Statistical analysis

Differences in age-adjusted mean values or the prevalence of potential confounding factors in relation to estimated daily salt intake levels were calculated using ANOVA or logistic regression models. Odds ratios (ORs) and 95 % confidence intervals (CI) for mental distress were calculated with the aid of logistic regression models. Two different approaches were used for making adjustments for confounding factors. First, we adjusted only for age, and, second, we included other potential confounding factors that included smoking status (never smoker, former smoker, current smoker), alcohol consumption (never drinker, former drinker, current drinker (<23 g/week, 23–46 g/week, 46–69 g/week, >69 g/week)), systolic blood pressure (mmHg), antihypertensive medication use (no/yes), thyroid disease (no/yes), body mass index (BMI) (kg/m^2^), taking glucose lowering medication (no/yes), HbA1c (%), HDL-cholesterol (mg/dL), TG (mg/dL), AST (IU/L), and GFR (mL/min/1.73 m^2^). It was previously reported that men with physical distress had a higher prevalence of high blood pressure than men without physical distress [12]. Since endocrine diseases such as hypothyroidism are known to be associated with hypotension and depressive symptoms, whereas high daily salt intake is positively associated with hypertension [1–4]; we included thyroid disease as a confounding factor. In our study, mental distress, which was evaluated only by K6 scale score, should only be classified by the status of hypertension. Therefore, we made an additional analysis that investigated the association between the estimated daily salt intake and mental distress with hypertension and mental distress without hypertension.

All statistical analyses were performed with the SAS system for Windows version 9.3 (SAS Inc., Cary, NC). Values of <0.05 were regarded as statistically significant.

## Results

Among the study population, 97 individuals were diagnosed as having mental distress. An age-adjusted study population according to tertiles of estimated daily salt intake is shown in Table 1. Estimated daily salt intake is significantly and positively correlated with BMI, systolic blood pressure, diastolic blood pressure, current drinker, and GFR while significantly and inversely correlated with current smoker.

**Table 1:** Age-adjusted clinical characteristics of the study population

Parameters	Estimated daily salt intake tertiles			*P* for trend
	T1 (low)	T2	T3 (high)	
No. at risk	338	338	338	
Age, year	65.6 ± 10.2	65.4 ± 10.1	64.3 ± 10.0	
Body mass index, kg/m^2^	23.0	23.4	24.1	<0.001
Systolic blood pressure, mmHg	132	136	137	<0.001
Diastolic blood pressure, mmHg	79	82	82	0.003
Antihypertensive medication use, %	40.2	38.7	36.8	0.640
Thyroid disease, %	1.5	1.2	0.9	0.813
Current drinker, %	75.4	77.5	85.3	0.002
Current smoker, %	26.4	18.5	18.4	0.012
Serum aspartate transaminase (AST), IU/L	26	25	25	0.226
Serum triglycerides, mg/dl	115	125	129	0.141
Serum HDL-cholesterol, mg/dl	56	54	56	0.184
HbA1C, %	5.7	5.6	5.7	0.363
Antidiabetic medication use, %	9.1	6.5	10.5	0.164
Glomerular filtration rate (GFR), mL/min/1.73 m^2^	68.1	69.7	74.1	<0.001
Kessler 6 score	1.6	1.3	1.2	0.151

Ages are given as mean ± standard deviation

### Estimated daily salt intake and mental distress

Table 2 shows the association between estimated daily salt intake and mental distress. Independent from classical cardiovascular risk factors and thyroid disease, we found a significant inverse association between estimated daily salt intake and mental distress. The multivariable odds ratios (ORs) of an increment of 1SD (standard deviation) in estimated daily salt intake (2.0 g/day) for mental distress was 0.74 (0.59–0.93).

**Table 2:** Odd ratios (OR) and 95 % confidence interval (CI) for mental distress in relation to estimated salt intake level tertiles

	Estimated daily salt intake tertiles			*P* for trend	1SD increment in estimated daily salt intake (2.0 g/day)
	Tl (low)	T2	T3 (high)		
No. at risk	338	338	338		
No. of cases (percentage)	45 (13.3)	29 (8.6)	23 (6.8)		
Age-adjusted OR	1.00	0.61 (0.37–1.00)	0.47 (0.28–0.79)	0.004	0.71 (0.57–0.88)
Multivariable OR	1.00	0.65 (0.39–1.09)	0.50 (0.29–0.88)	0.014	0.74 (0.59–0.93)

Multivariable OR: adjusted further for body mass index, systolic blood pressure, antihypertensive medication use, thyroid disease, alcohol consumption, smoking status, AST, triglycerides, HDL-cholesterol, HbA1c, antidiabetic medication use, and GFR. The median values of estimated daily salt intake tertiles were 7.0, 8.8, and 10.8 g/day

### Kessler 6 (K6) and blood pressure

We also evaluated the influence of the K6 scale score on blood pressure. We found the K6 scale score was significantly and inversely correlated with systolic blood pressure whereas no significant correlation was observed for diastolic blood pressure. By age-adjusted linear regression analysis, the parameter estimates were β = −0.42 (P = 0.036) for systolic blood pressure and β = −0.08 (P = 0.554) for diastolic blood pressure.

### Estimated daily salt intake and mental distress classified by status of hypertension

Table 3 shows the associations between estimated daily salt intake and mental distress classified by levels of hypertension. For mental distress with hypertension, a significant inverse association was observed with estimated daily salt intake. And for mental distress without hypertension, essentially the same association was observed, although the association did not reach statistical significance. With the reference group as the lowest tertile of estimated daily salt intake, the multivariable ORs of mental distress with hypertension and mental distress without hypertension for the highest tertile of estimated daily salt intake were 0.46 (0.22–0.96) and 0.48 (0.20–1.16), respectively.

**Table 3:** Odds ratios (OR) and 95 % confidence interval (CI) for mental distress classified by hypertension status in relation to estimated salt intake level tertiles

	Estimated daily salt intake tertiles			*P* for trend	1SD increment in estimated daily salt intake (2.0 g/day)
	T1 (low)	T2	T3 (high)		
No. at risk	338	338	338		
Mental distress with hypertension					
No. of cases (percentage)	26 (7.7)	21 (6.2)	12 (3.6)		
Age-adjusted OR	1.00	0.80 (0.44–1.45)	0.45 (0.22–0.90)	0.025	0.76 (0.58–0.99)
Multivariable OR	1.00	0.86 (0.46–1.60)	0.46 (0.22–0.96)	0.045	0.77 (0.58–1.03)
Mental distress without hypertension					
No. of cases (percentage)	19 (5.6)	8 (2.4)	11 (3.3)		
Age-adjusted OR	1.00	0.40 (0.17–0.93)	0.53 (0.25–1.14)	0.082	0.67 (0.48–0.94)
Multivariable OR	1.00	0.42 (0.17–1.05)	0.48 (0.20–1.16)	0.090	0.69 (0.47–1.02)

Multivariable OR: adjusted further for body mass index, systolic blood pressure, antihypertensive medication use, thyroid disease, alcohol consumption, stroking status, AST, triglycerides. HDI-cholesterol, HbAlc, antidiabetic medication use, and GFR. The median values of estimated daily salt intake tertiles were 7.0, 8.8, and 10.8 g/day

### Body mass index and mental distress classified by the levels of hypertension

Depressive symptoms might cause appetite loss and result in lower BMI. We also evaluated the influence of BMI on mental distress with and without hypertension. The BMI showed no significant association with mental distress with hypertension, but there was a significant inverse association observed for mental distress without hypertension. The age-adjusted ORs with an increment of 1SD in BMI (3.1 kg/m^2^) for mental distress with hypertension and without hypertension were 1.08 (0.83–1.40, *P* = 0.586) and 0.64 (0.46–0.91, *P* = 0.013), respectively.

## Discussion

The main finding of the present study is that independent from classical cardiovascular risk factors, estimated daily salt intake is inversely associated with mental distress. This is especially true for mental distress with hypertension among rural community-dwelling Japanese men.

The RAS-related C3 botulinum toxin substrate 1 (Rac1) and mineralocorticoid receptor might be important underlying factors. Several lines of evidence indicate that an impaired feedback regulation between sodium and aldosterone/mineralocorticoid receptors appears to impact the salt-induced hypertension and cardiorenal damage [13–15]. A recent study by Shibata et al. identified signaling cross-talk between mineralocorticoid receptors and the small GTPase Rac1 as a novel pathway that modulates mineralocorticoid receptor function [3]. In their study, a high-salt intake acted synergistically with aldosterone to activate renal Rac1 in salt-sensitive hypertension loading, resulting in high blood pressure and renal damage through potentiating mineralocorticoid receptors. The variable response of renal Rac1 to high-sodium loading was a key mechanism that modulates the salt sensitivity of blood pressure and kidney injury [3]. Rac1 GTPase is involved in the heterogeneity of salt sensitivity through mineralocorticoid receptor modulation. The study demonstrates that high-salt loading triggers Rac1 activation in the kidneys in salt-sensitive hypertension, which is the key event in the pathogenesis of blood pressure elevation and kidney injury via a mineralocorticoid receptor-dependent pathway. In our study, among all subjects, the estimated daily salt intake level is significantly and positively correlated with blood pressure levels, for systolic and diastolic blood pressure. Then, estimated daily salt intake might indicate the activity of Rac1 and the mineralocorticoid receptors.

The mechanisms underlying the inverse association between estimated daily salt intake and mental distress are not yet clarified. A sustained reduction in Rac1 expression after chronic social defeat stress has also been reported in a previous study [16], and another study revealed gestational stress and excess maternal plasma corticosterone cause downregulation of mineral corticoid receptors in rats [17]. Individuals with relatively low mineralocorticoid receptor function may have increased susceptibility to depression in response to stress [6]. These studies suggested to us that estimated daily salt intake may be inversely associated with mental distress underlying reduced Rac1 activity and mineralocorticoid receptor activity. In addition, since Rac1 GTPase and mineral corticoid receptors are activated in salt-induced hypertension [3], the mental distress patient with hypertension and lower daily salt intake might have a lower activity of Rac1 GTPase and mineral corticoid receptors. We also observed a significant inverse association between the estimated daily salt intake and mental distress with hypertension, whereas men with physical distress were reported to have a higher prevalence of high blood pressure than do those without physical distress [12]. In our additional analysis, even with no significant correlation between estimated daily salt intake and blood pressure (either for systolic and diastolic) for mental distress patients with hypertension (*N* = 59), we found a significant positive correlation for mental distress patients without hypertension (*N* = 38). By age-adjusted linear regression analysis, the parameter estimates for patients with mental distress with hypertension were β = 0.59 (P = 0.672) for systolic blood pressure and β = 0.55 (P = 0.574) for diastolic blood pressure, and for mental distress patients without hypertension the corresponding values were β = 2.77 (P = 0.045) and β = 2.13 (P = 0.015), respectively. Therefore, we hypothesize that the mechanisms of the hypertension for mental distress patients are different from the mechanism of salt-induced hypertension.

There are potential limitations of our study that warrant consideration. Since we have no data on mineralocorticoid receptor activity or on Rac 1 activity, the exact mechanism is not yet clear. Further investigations that include those data are necessary. Second, depression symptoms might cause appetite loss that could also cause low daily salt intake. However in our study, even after we adjusted further for BMI, the association between estimated daily salt intake and mental distress remained significant. Furthermore, we also evaluated the influence of BMI on mental distress with and without hypertension, and we found BMI is significantly and inversely associated with mental distress without hypertension, but no significant association was observed for mental distress with hypertension. Since we did not have access to depression treatment data, we could not determine the possible effect of depression treatment on both the estimated daily salt intake and the mental distress. Further investigations with depression treatment data are necessary.

Finally, because this was a cross-sectional study, no causal relationships could be established.

In conclusion, independent from classical cardiovascular risk factors, the estimated daily salt intake is inversely associated with mental distress, especially for men with mental distress with hypertension among rural community-dwelling Japanese. Since depression is reported to be associated with cardiovascular disease [7], the risk estimation for mental distress associated with salt intake may become an important tool to prevent cardiovascular disease.

## Abbreviations

24HuNaV: estimated population mean levels of 24-h urinary sodium
AST: aspartate transaminase
BMI: body mass index
CI: confidence intervals
GFR: glomerular filtration rate
HbA1c: hemoglobin A1c
K6: Kessler 6
ORs: odds ratios
Rac 1: RAS-related C3 botulinum toxin substrate 1
ROC: receiver operating characteristic
PRCr: predicted values of 24-h urinary creatinine
SD: standard deviation
SUCr: creatinine concentration in the spot voiding urine
SUNa: Na concentration in the spot voiding urine
TG: triglycerides
XNa: SUNa/SUCr×PRCr
